# PRIMARY LANGUAGE’S ROLE IN SOCIAL INTEGRATION AND FUNCTIONAL INDEPENDENCE AFTER TRAUMATIC BRAIN INJURY

**DOI:** 10.1093/geroni/igae098.4054

**Published:** 2024-12-31

**Authors:** Wonkyung Jung, Janiece Taylor

**Affiliations:** Boston College, Chestnut Hill, Massachusetts, United States; Johns Hopkins University, Johns Hopkins University, Maryland, United States

## Abstract

Social integration is an ultimate goal of neurorehabilitation and linked to better functional outcomes. However, older immigrants whose primary language is not English may have difficulties reintegrating into society after their injuries. This study investigated the moderating role of primary language in the relationship between social integration and functional independence following traumatic brain injury (TBI) among older adults at the first-year post-injury. Data were obtained from the Traumatic Brain Injury Model System National Database (TBIMS-NDB). Using Hayes’s PROCESS macro-Model 1, data were analyzed to assess the impact of social integration and primary language on functional independence. We finally had 1442 older adults aged between 65 and 88 years at the time of TBI. The results revealed that social integration significantly influenced functional independence (β = 11.399, p < 0.001), while primary language had an insignificant impact (β = -3.735, p = 0.051). Additionally, a significant interaction effect was found between social integration and language (β = 6.912, p = 0.006), indicating that the relationship between social integration and functional independence was moderated by primary language. The model explained a substantial proportion of the variance in functional independence (R-Square = 0.328). Further analysis showed that the effect of social integration on functional independence varied based on language proficiency, with a stronger association observed among non-native English speakers (β = 25.224) compared to native English speakers (β = 18.311). These findings highlight the need to consider primary language in interventions to improve social integration and functional outcomes for older adults with TBI.

